# A Rare Case of Severe Hypercalcemia Secondary to Atypical Parathyroid Cystic Adenoma with Negative Sestamibi Scan

**DOI:** 10.7759/cureus.6830

**Published:** 2020-01-31

**Authors:** Elio P Monsour, Faysal Rifai, Jay Chacko, Alan Hamza, Khalid Abusaada

**Affiliations:** 1 Internal Medicine, Ocala Regional Medical Center/ University of Central Florida College of Medicine, Ocala, USA

**Keywords:** hypercalcemia, primary hyperparathyroidism, parathyroid sestamibi scan, parathyroid adenoma

## Abstract

The two types of parathyroid cysts are functional and non-functional cysts. Cystic parathyroid lesions are a rare cause of hypercalcemia and often pose a diagnostic challenge due to the reduced detection on preoperative imaging studies. We, herein, present a rare case of an elderly female presenting to the emergency department with altered mental status associated with hypercalcemic crisis and a negative sestamibi scan. Following surgical resection, pathology revealed the diagnosis of cystic parathyroid adenoma and normalization of serum calcium levels.

## Introduction

Primary hyperparathyroidism (PHPT) is defined as excessive secretion of parathyroid hormone (PTH) originating from the parathyroid gland. PHPT is most commonly caused by a single parathyroid adenoma, in which the majority of lesions tend to be solid. Cystic parathyroid adenomas, however, are an infrequent entity and are seen in approximately 1-2 % of all patients with primary hyperparathyroidism [1]. Cystic parathyroid adenomas are also known as functional parathyroid cyst (FPC) and are said to be due to cystic degeneration of an existing parathyroid adenoma [2]. We describe an unusual case of severe hypercalcemia secondary to 99mTc sestamibi scan negative atypical parathyroid cystic adenoma in a patient presenting with altered mental status and generalized weakness. 

## Case presentation

**Figure 1:**
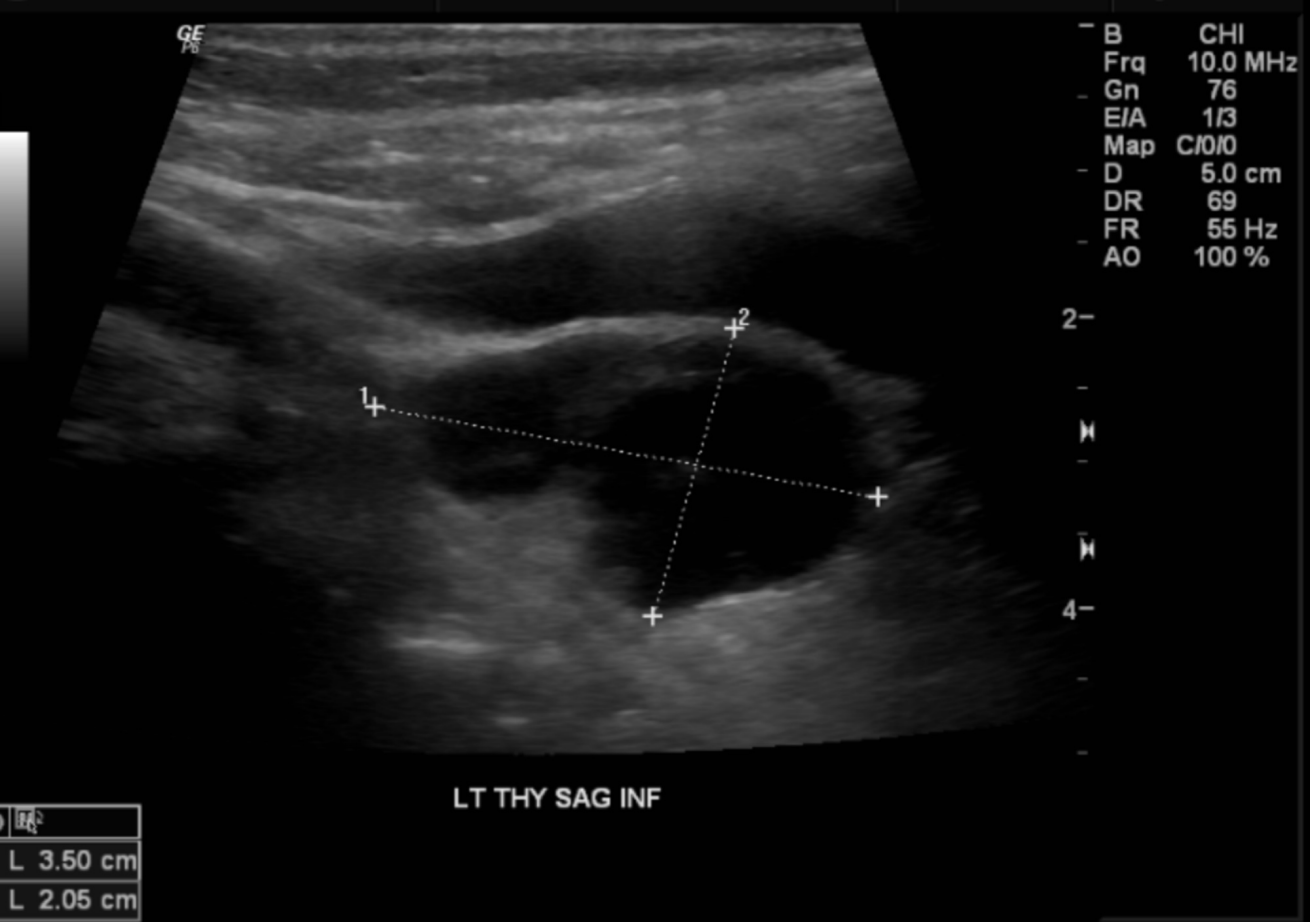
Thyroid and parathyroid ultrasound revealing a predominantly cystic appearing mass with septations and mild irregularity measuring roughly 3.5 x 2.1 x 2.0 cm

A 69-year-old female presented to our facility with severe lethargy and altered mental status. Her past medical history included osteopenia on calcium and vitamin D supplements, diet-controlled diabetes mellitus, and hypertension. She also had a recent mechanical ground level fall and surgically treated hip fracture one month prior to presentation. -10.5), ionized calcium of 7. On physical exam, the patient was arousable but unable to follow commands. Further examination findings included decreased skin turgor and dry oral mucosa. Upon admission, the patient was afebrile with a blood pressure of 165/79 mmHg, a heart rate of 109, a respiratory rate of 20 breaths per minute, and oxygen saturation of 96 % on ambient air. Chemistry and serology showed a serum calcium of 20.4 mg/dL (8.88 mg/dL (3.8-4.8), phosphorus of 2.1 mg/dL (2.5-4.9), magnesium of 1.4 mg/dL (1.8-2.5 mg/dL), intact PTH of 731.8 pg/mL(<65), PTH-related peptide <2.0 pmol/L (<2.0 pmol/L), creatinine of 1.10 mg/dL, 25-OH vitamin D of 34.5 ng/mL, 1,25 di-OH vitamin D of 87.5 pg/dL(19.9−79.3 pg/mL), alkaline phosphatase of 183 IU/L (38-126 IU/L), and 24-h urine calcium of 200 mg/24 h (100.0−300.0). Imaging included parathyroid ultrasonography, which revealed a cystic appearing mass with septations and mild irregularity measuring 3.5 x 2.1 x 2.0 cm in the inferior aspect of the left lobe of the thyroid gland (Figure 1).

**Figure 2:**
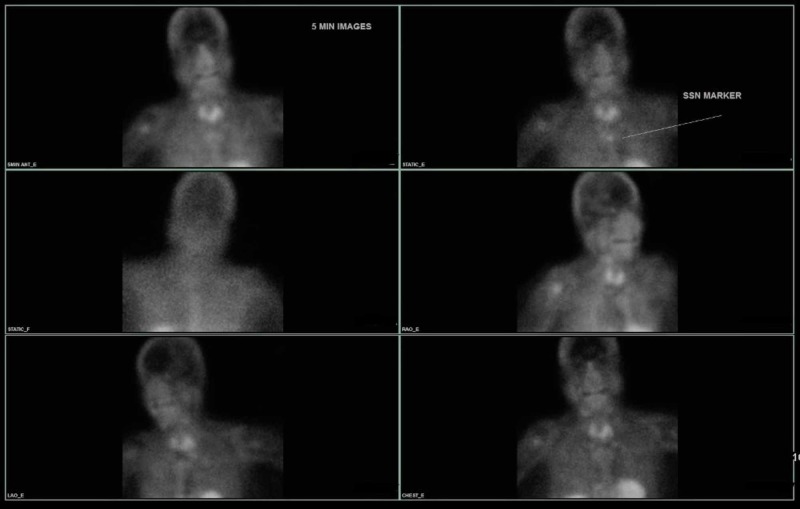
99 mTc Sestamibi parathyroid scan demonstrating no abnormal or residual radiotracer activity to suggest parathyroid adenoma

**Figure 3:**
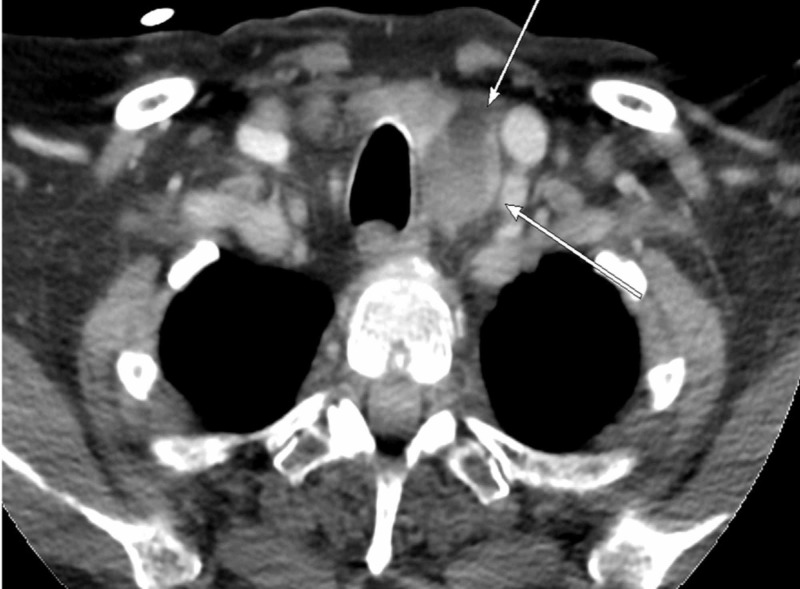
Axial section of parathyroid 4 dimension (4D) computed tomography (CT) demonstrating abnormal mass abutting the posterior left aspect of the thyroid gland representing a parathyroid adenoma

She was started on aggressive intravenous hydration, calcitonin 4 units/kg, 2 mg of IV zoledronic acid, and cinacalcet 60 mg once daily. Upon stabilization, parathyroid sestamibi scan was conducted but was negative (Figure 2). Further workup included a sestamibi scan of the abdomen/pelvis, which was unremarkable for ectopic parathyroid secreting tumor. Parathyroid four-dimensional (4D) computed tomography (CT) scan was performed and showed an abnormal mass abutting the left posterior aspect of the thyroid gland, possibly representing a parathyroid adenoma (Figure 3).

 

**Figure 4:**
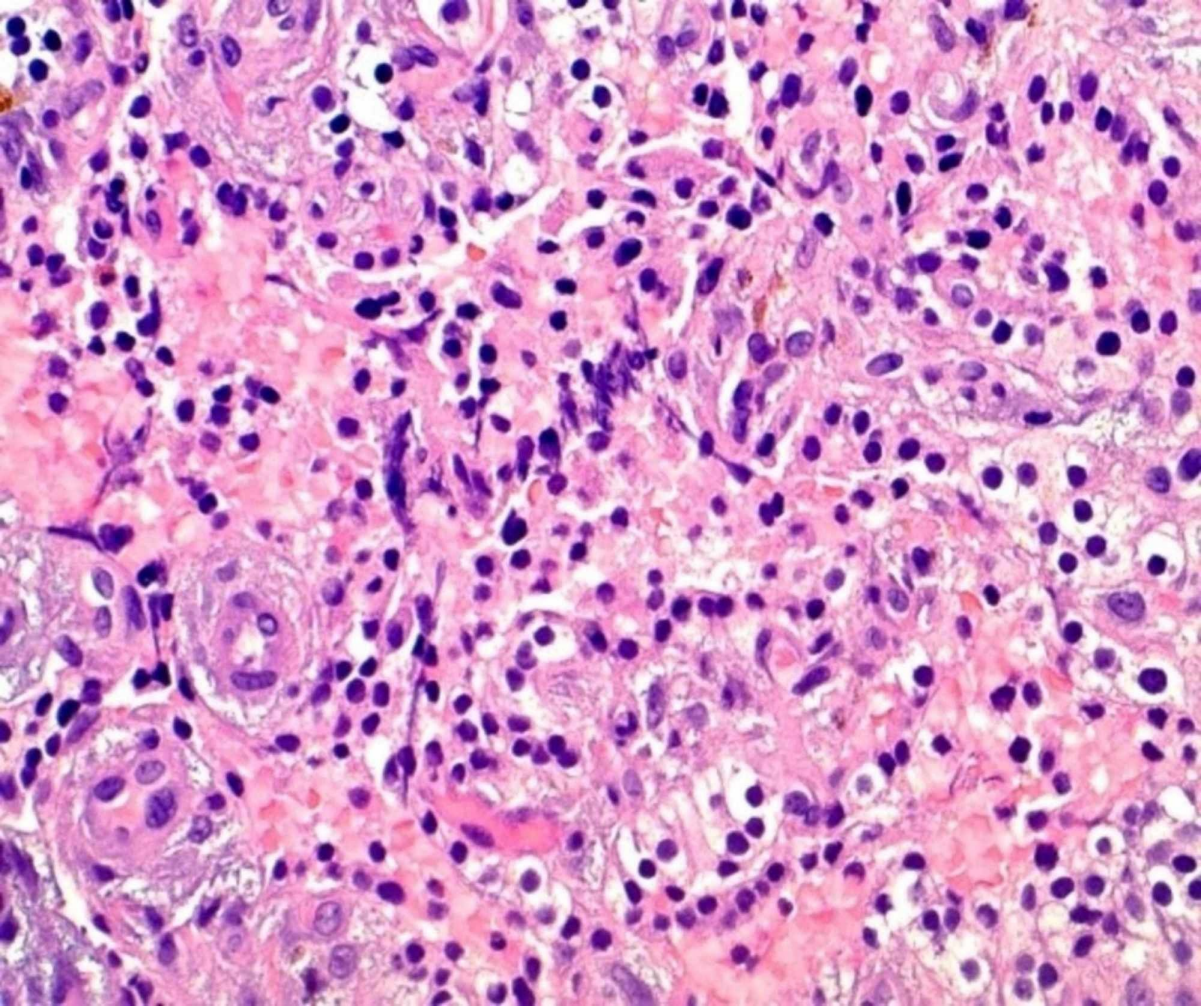
Photomicrograph of parathyroid gland using hematoxylin and eosin staining (approximate magnification 20x) revealed cystic parathyroid tissue with focal atypia, cystic degeneration with hemorrhage

Her mental status improved over the following days as rehydration continued, and repeat calcium levels began to downtrend. Otolaryngology service was consulted for parathyroidectomy with possible neck exploration. The patient underwent left inferior parathyroidectomy, and intraoperative frozen section analysis was read as a cystic parathyroid adenoma. The final pathology report revealed cystic parathyroid tissue favoring parathyroid adenoma with focal atypia (Figure 4). Immunohistochemical staining with Ki-67 shows approximately 3-4% positivity, supporting the diagnosis of atypical parathyroid adenoma. Following parathyroidectomy, intact PTH levels were found to have decreased to 7.5 pg/mL, and calcium levels decreased to 11.3. The patient was closely monitored, improved significantly, and was started on calcium carbonate tablets 1000 mg three times daily. Her final ionized calcium was 4.0 mg/dl.

## Discussion

Atypical cystic parathyroid adenomas are a rare cause of primary hyperparathyroidism (PHPT) and account for less than 5 % of all parathyroid adenomas. On the other hand, 90% of parathyroid cysts were often associated with normocalcemia and were nonfunctional [3]. In a study conducted in China, patients with cystic parathyroid adenomas were more likely to present with hypercalcemic crisis than the solid adenoma group, and the overall predominance of cystic adenomas was higher in males [3]. Furthermore, in contrast to solid adenomas, patients with cystic parathyroid adenomas had higher preoperative serum iPTH and calcium levels along with lower accuracy in diagnostic imaging techniques such as neck ultrasonography and 99mTc sestamibi scan [3]. However, in cases in which ultrasound and 99mTc sestamibi scans were negative, parathyroid 4-dimensional computed tomography (CT) may be able to identify up to 80% of abnormal glands [4]. 

The pathogenesis of atypical cystic parathyroid adenomas is thought to be secondary to cystic degeneration of pre-existing parathyroid adenomas [3]. This phenomenon possibly explains the abrupt rise in intact PTH in circulation, which can trigger a hypercalcemic crisis. Further contributions to severe hypercalcemia in our case described above can also be possibly attributed to immobilization secondary to recent orthopedic surgery, and use of calcium and vitamin D supplementation. Generally speaking, the overall prognosis of cystic lesions is excellent with surgical excision, and the recurrence rate is minimal with a proper technique [5]. 

## Conclusions

We report a compelling case of a patient presenting with hypercalcemic crisis secondary to cystic parathyroid adenoma, which posed a diagnostic challenge as both neck ultrasound and 99mTc sestamibi scan were inconclusive. These findings should trigger clinical suspicion for functional parathyroid cystic lesions. In such cases, a 4D parathyroid CT scan may provide additional diagnostic information and adenoma localization. Nonetheless, intraoperative 99mTc sestamibi as well as iPTH assay assisted in localizing the functioning lesion, which was surgically resected, and ultimately curative. 

## Appendices

“This research was supported (in whole or in part) by HCA Healthcare and/or an HCA Healthcare affiliated entity. The views expressed in this publication represent those of the author(s) and do not necessarily represent the official views of HCA Healthcare or any of its affiliated entities.” 
